# Supplementary figures and images for: Application of biochar from crop straw in asphalt modification
Source: PLoS One. 2021 Feb 25;16(2):e0247390. doi: 10.1371/journal.pone.0247390 (PMC7906466; doi:10.1371/journal.pone.0247390)

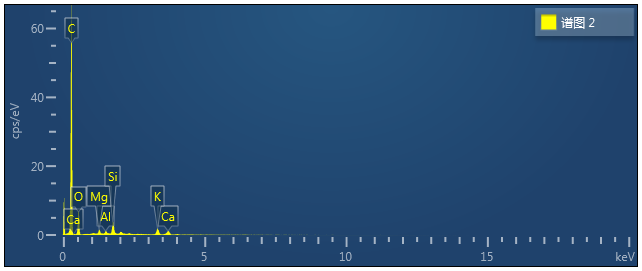

Supplement: S1 Fig — (TIF) [file pone.0247390.s001.tif]

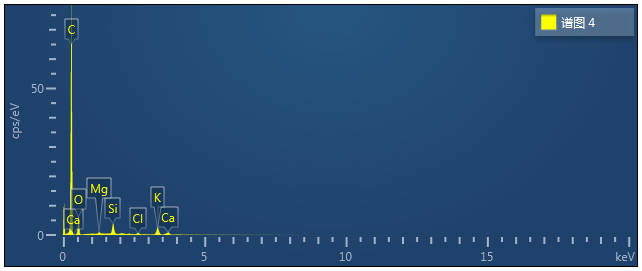

Supplement: S2 Fig — (TIF) [file pone.0247390.s002.tif]
