# Supplementary material for: Application of biochar from crop straw in asphalt modification
Source: PLoS One. 2021 Feb 25;16(2):e0247390. doi: 10.1371/journal.pone.0247390 (PMC7906466; doi:10.1371/journal.pone.0247390)
Supplement: Attachment — Submitted filename: PLOS_ONE.docx [file pone.0247390.s003.docx]

In this manuscript, the authors added bio-char to an asphalt mixtures and assessed the microscopic and physical properties associated with this new recipe. This is an interested topic, but unfortunately it presents like a case study/technical note and less like a full-length journal article because the analysis is relatively unsophisticated and there is little to no discussion. While I am perfectly capable of connecting the dots myself, the authors should do this for the reader. In addition, it took the authors’ significant time to “get going” in the sense that for the first several sections there were grammatical or punctuation errors in almost every sentence. My line-by-line comments are therefore extensive and presented below. I recommend that the authors be given an opportunity for a revision, but much more sophisticated analysis will be required if this paper has any hope of being published.

Introduction

- First paragraph, 3^rd^ line from bottom – I do not think “rational” is the word you want there. Suggest replacing with “sustainable.”
- First paragraph, sentence starting with “One possible method….” This is a sentence fragment/improper tenses/punctuation. It needs to be rewritten.
- Second paragraph, first sentence – remove the words “owing to repeated usage.” They are unnecessary, and the sentence should just start with “The performance of asphalt pavements….”
- Second paragraph, sentence that starts with “Under” – the word “owing” is used incorrectly. You mean “because of”
- Next sentence, remove “Being a cementitious material”
- Sentence after that, remove the words “Therefore, owing” and replace with “Because of”
- Third paragraph – extra “the” before adding.
- Third paragraph, third line – remove the comma after mixtures

Lit Review

- 4th line – remove the word “purposes” after “energy generation;” remove the dashes around the “such as” clause and replace with commas.
- Sentence that starts with S.M. Shafie – remove the comma after “logistical costs.”
- Same sentence and next sentence – inconsistent formatting with your references. Above you were referring to your references by last name only. Then you did initials and last name. And here it is full first name and last name (Mingxing Sun). And then in the next sentence you are back to last name only (Nair). Which is correct? Be consistent and stick to it!
- Next paragraph – same inconsistencies noted above (Zhao Sheng or just “Sheng” would probably be more correct); Renaldo Walters vs.”Walters.” Etc.
- Sentence that starts with “Renaldo Walters” – you wrote 3 and 6%. The correct punctuation is “3% and 6%.”
- Sentence starting with “FU Zhen” – this sentence does not make any sense unless you add the word “at” before the word “were.” (i.e. …asphalt that were conducted at….”

Materials and Methods

- Sentence right after Fig. 2 – this should be in the past tense. I.e., the preparation process was as follows.
- Step 4 – the subject of this sentence is “the straws.” Therefore, the correct pronoun to refer to this subject is “they” and not “it.”
- Section 4.3 – What are the details associated with your physical property testing? Were there repetitions? If so, how many? Was a standard followed? If so, it must be referenced.

Results and Discussion

- Figs. 3 & 4 – I can see from the figure that the authors did not bother to make a “clean” document for submission and instead left the track changes on in MS Word. This is sloppy/one should expect better than this if this paper is truly ready for publication.
- Section 5.2 – third paragraph through the end of this section – I can clearly see the point the authors are making – bio-char has much smaller particles statistically than commercial-grade charcoal. However, I find myself wondering if this is the best way to present these data. Would it not be more efficient/concise to express Table 4 in graphical form similar to an aggregate particle distribution chart or a geotechnical soil particle chart? I would think this would make the data a little bit easier to read/interpret. In either case, remove the word “owing” in the 3^rd^ to last line from the bottom of this section. Almost always, you can strike “owing” from your writing and either remove it entirely or replace it with the word “because.”
- Section 5.3 – remove the word “moreover” from the sentence that starts with “As the biochar content increased…” Also add semicolon before “and the ductility”
- Same sentence in Section 5.3 – you said that bio-char “significantly increased high-temperature performance.” Be careful with the word significantly unless you really mean it. It does not appear that there was any testing repetition, although based upon Section 4.3, it is very difficult to tell exactly what the authors did. If there were no repetitions, it would be very difficult to prove statistical significance. If the authors would be willing to couch their argument to something less definitive (i.e., remove the word “significant”) this would be fine as well. In either case, I would think a graphical presentation might be a better way to present these data as opposed to putting them in tabular form. Doing so would allow you to do much more interesting analysis like (for example) plotting a regression line through the data points. This does not prove statistical significance, but it does show a relationship between % biochar and physical properties and would be much more sophisticated than what is presented here. I just plotted the data myself and noted that there appears to be a linear relationship between softening and % bio-char; and apparent exponential relationships between penetration & density and % bio-char. Each of these relationships had relatively high R-squared values.

Conclusions

The authors have dutifully summarized there work here. However, they have done almost nothing to connect the dots for the reader. In other words, what do these results imply for future asphalt mixtures? Might they be applicable to other types of asphalt with different aggregate? Would these results mean that this would be a good idea or a bad idea for future roadway projects? The authors appear to imply “bad idea,” and the null answer is not necessarily unpublishable. But, the discussion/conclusion reads more like a “data dump” and less like a paper. Much more sophisticated analysis is needed to tell the reader why we should care about this/what the results mean.
