# Supplementary material for: Application of biochar from crop straw in asphalt modification
Source: PLoS One. 2021 Feb 25;16(2):e0247390. doi: 10.1371/journal.pone.0247390 (PMC7906466; doi:10.1371/journal.pone.0247390)
Supplement: Attachment — Submitted filename: Response to reviewers.doc [file pone.0247390.s004.doc]

Dear Editor,

We are so appreciated for your letter on our manuscript (PONE-D-20-24402), entitled “Application of Bio-char from Crop Straw in Asphalt Modification” . We are also extremely greatful to the reviewers’ comments on our manuscript and carefully considered every comment, and made cautious revision accordingly. Based on their suggestions, we have answered the questions in detail one by one. If you have any other questions about this paper, I would quite appreciate it if you could let me know them in the earliest possible time.

Most sincerely,

Xinli Gan

November 25, 2020

**Additive list**

**Introduction :**

**Comment 1:** First paragraph, 3rd line from bottom – I do not think “rational” is the word you want there. Suggest replacing with “sustainable.”

**Answer:** Thanks for your very thoughtful suggestion. We have revised this sentence as follows: “Therefore, a sensible and sustainable way of using crop straw has become an important research and real-world application issue.”

**Comment 2:** First paragraph, sentence starting with “One possible method….” This is a sentence fragment/improper tenses/punctuation. It needs to be rewritten.

**Answer:** Thanks for your very thoughtful suggestion. We have revised this sentence as follows: “Therefore, a sensible and sustainable way of using crop straw has become an important research and real-world application issue.”
**Comment 3:** Second paragraph, first sentence – remove the words “owing to repeated usage.” They are unnecessary, and the sentence should just start with “The performance of asphalt pavements….”

**Answer:** Thanks for your very thoughtful suggestion. We have revised this sentence as follows: “Simultaneously, during asphalt pavement operation, its performance inevitably declines under the combined action of load and the natural environment.”

**Comment 4:** Second paragraph, sentence that starts with “Under” – the word “owing” is used incorrectly. You mean “because of”

**Answer:** Thanks for your very thoughtful suggestion. We have revised this sentence as follows: “Simultaneously, during asphalt pavement operation, its performance inevitably declines under the combined action of load and the natural environment.”

**Comment 5:** Next sentence, remove “Being a cementitious material”

**Answer:** Thanks for your very thoughtful suggestion. We have revised this sentence as follows: “Asphalt, as a cementitious material, ages under the comprehensive use of light and temperature, which reduce its cementation ability and negatively affect the durability of asphalt pavement.”

**Comment 6:** Sentence after that, remove the words “Therefore, owing” and replace with “Because of”

**Answer:** Thanks for your very thoughtful suggestion. We have revised this sentence as follows: “Owing to its high cost and environmental sensitivity, improving the durability of asphalt pavement has become the focus of road workers.”

**Comment 7:** Third paragraph – extra “the” before adding.

**Answer:** Thanks for your very thoughtful suggestion. We've added "the" before "adding"

**Comment 8:** Third paragraph, third line – remove the comma after mixtures.

**Answer:** Thanks for your very thoughtful suggestion. We've removed the comma after “mixtrues”, and revised this sentence as follows: “and aging resistance of asphalt and asphalt mixtures and does not adversely affect other asphalt pavement performance indicators .”

**Lit Review :**

**Comment 1:** 4th line – remove the word “purposes” after “energy generation;” remove the dashes around the “such as” clause and replace with commas.

**Answer:** Thanks for your very thoughtful suggestion. We have revised this sentence as follows: “studied the economic gains from using it as a fuel, as raw material for papermaking, and as animal feed, compared to burning the straw in a field. Smuga-Kogut et al.”

**Comment 2:** Sentence that starts with S.M. Shafie – remove the comma after “logistical costs.”

**Answer:** Thanks for your very thoughtful suggestion. We have removed the comma after “logistical costs.”

**Comment 3:** Same sentence and next sentence – inconsistent formatting with your references. Above you were referring to your references by last name only. Then you did initials and last name. And here it is full first name and last name (Mingxing Sun). And then in the next sentence you are back to last name only (Nair). Which is correct? Be consistent and stick to it!

**Answer:** Thanks for your very thoughtful suggestion. We have revised the format of the references.

**Comment 4:** Next paragraph – same inconsistencies noted above (Zhao Sheng or just “Sheng” would probably be more correct); Renaldo Walters vs.”Walters.” Etc.

**Answer:** Thanks for your very thoughtful suggestion. We have revised the format of the references.

**Comment 5:** Sentence that starts with “Renaldo Walters” – you wrote 3 and 6%. The correct punctuation is “3% and 6%.”

**Answer:** Thanks for your very thoughtful suggestion. We have added "%" after "3"

**Comment 6:** Sentence starting with “FU Zhen” – this sentence does not make any sense unless you add the word “at” before the word “were.” (i.e. …asphalt that were conducted at….”

**Answer:** Thanks for your very thoughtful suggestion. We have revised this sentence as follows: “ Zhen et al. prepared bio-char-modified asphalt via the high-speed shear method and conducted laboratory RTFOT and 60 °C dynamic viscosity tests of the bio-char-modified asphalt; they found that bio-char could strengthen the bond capability and resistance to flow deformation of the modified asphalt.”

**Materials and Methods :**

**Comment 1:** Sentence right after Fig. 2 – this should be in the past tense. I.e., the preparation process was as follows.

**Answer:** Thanks for your very thoughtful suggestion. We have revised this sentence as follows:”The preparation process was as follows”

**Comment 2:** Step 4 – the subject of this sentence is “the straws.” Therefore, the correct pronoun to refer to this subject is “they” and not “it.”

**Answer:** Thanks for your very thoughtful suggestion. We have revised this sentence as follows:”After being cooled, they were ground to a powder using a grinder.”

**Comment 3:** Section 4.3 – What are the details associated with your physical property testing? Were there repetitions? If so, how many? Was a standard followed? If so, it must be referenced.

**Answer:** Thanks for your very thoughtful suggestion. The standard of these tests followed is the Standard Test Methods of Bitumen and Bituminous Mixtures for Highway Engineering (JTG E20-2011) of China. It has been supplemented in the manuscript. The number of repetitions of each test was carried out according to the Standard Test Methods of Bitumen and Bituminous Mixtures for Highway Engineering (JTG E20-2011) of China. The ductility test was repeated three times in each group, the softening point test was repeated twice in each group, the penetration test was repeated three times in each group.

**Results and Discussion :**

**Comment 1:** Figs. 3 & 4 – I can see from the figure that the authors did not bother to make a “clean” document for submission and instead left the track changes on in MS Word. This is sloppy/one should expect better than this if this paper is truly ready for publication.

**Answer:** Thanks for your very thoughtful suggestion. We are very sorry for these problems because we didn't proofread them carefully. We have proofread the manuscript again.

**Comment 2:** Section 5.2 – third paragraph through the end of this section – I can clearly see the point the authors are making – bio-char has much smaller particles statistically than commercial-grade charcoal. However, I find myself wondering if this is the best way to present these data. Would it not be more efficient/concise to express Table 4 in graphical form similar to an aggregate particle distribution chart or a geotechnical soil particle chart? I would think this would make the data a little bit easier to read/interpret. In either case, remove the word “owing” in the 3^rd^ to last line from the bottom of this section. Almost always, you can strike “owing” from your writing and either remove it entirely or replace it with the word “because.”

**Answer:** Thanks for your very thoughtful suggestion. Because the particle size of carbon powder is very small (micron size), it is difficult to characterize it by the grading distribution diagram. Therefore, in this article, a laser particle size analysis test of the crop straw bio-char and commercial charcoal was conducted to evaluate the particle size characteristics of carbon powder. In addition, we replaced the word “owing” with the word “because.”

**Comment 3:** Section 5.3 – remove the word “moreover” from the sentence that starts with “As the biochar content increased…” Also add semicolon before “and the ductility”

**Answer:** Thanks for your very thoughtful suggestion. We have revised this sentence as follows:“It was found that, with the increase in bio-char content, the asphalt penetration decreased, the softening point increased, and the ductility decreased. ”

**Comment 4:** Same sentence in Section 5.3 – you said that bio-char “significantly increased high-temperature performance.” Be careful with the word significantly unless you really mean it. It does not appear that there was any testing repetition, although based upon Section 4.3, it is very difficult to tell exactly what the authors did. If there were no repetitions, it would be very difficult to prove statistical significance. If the authors would be willing to couch their argument to something less definitive (i.e., remove the word “significant”) this would be fine as well. In either case, I would think a graphical presentation might be a better way to present these data as opposed to putting them in tabular form. Doing so would allow you to do much more interesting analysis like (for example) plotting a regression line through the data points. This does not prove statistical significance, but it does show a relationship between % biochar and physical properties and would be much more sophisticated than what is presented here. I just plotted the data myself and noted that there appears to be a linear relationship between softening and % bio-char; and apparent exponential relationships between penetration & density and % bio-char. Each of these relationships had relatively high R-squared values.

**Answer:** Thanks for your very thoughtful suggestion.

Many studies have shown that the softening point, penetration and ductility of asphalt are significantly related to the high temperature performance of asphalt. The better the high temperature performance of asphalt, the higher the softening point, the smaller penetration and ductility. The standard of these tests followed is the Standard Test Methods of Bitumen and Bituminous Mixtures for Highway Engineering (JTG E20-2011) of China. Each test was repeated in accordance with the requirements of the standard.

**Conclusions :**

**Comment 1:** The authors have dutifully summarized there work here. However, they have done almost nothing to connect the dots for the reader. In other words, what do these results imply for future asphalt mixtures? Might they be applicable to other types of asphalt with different aggregate?

**Answer:** Thanks for your very thoughtful suggestion. We rewrite the conclusion as follows: “It is feasible to prepare bio-char from crop straw and add it into asphalt, especially in high temperature areas. Although the biological addition of crop straw reduces the low-temperature performance of asphalt, for high-temperature areas, there is almost no road low-temperature disease; thus, the requirement of asphalt low-temperature performance is very low. The addition of crop straw bio-char increases the high temperature performance of asphalt, which is beneficial to asphalt pavement in high temperature areas. In addition, some studies have shown that adding commercial charcoal in the form of carbon powder can improve the high temperature performance and fatigue performance of asphalt. The elements in the crop straw bio-char are mainly C, O, Si, and K, which is consistent with the chemical composition of commercial charcoal. This indicates that the bio-char from crop straw has a similar effect. In addition, through the experimental study, this article recommends that the appropriate amount of straw bio-char powder used in asphalt is 6 %, and the construction temperature should be appropriately increased.”
