# Supplementary material for: Application of biochar from crop straw in asphalt modification
Source: PLoS One. 2021 Feb 25;16(2):e0247390. doi: 10.1371/journal.pone.0247390 (PMC7906466; doi:10.1371/journal.pone.0247390)
Supplement: Attachment — Submitted filename: Response_to_reviewers.doc [file pone.0247390.s006.doc]

[January 28, 2021]

Dear Editor:

I wish to re-submit the manuscript titled “Application of biochar from Crop Straw in Asphalt Modification” for publication in PLOS ONE. The manuscript ID is PONE-D-20-24402R2.

I thank you and the reviewers for your thoughtful suggestions and insights. The manuscript has benefited from these insightful suggestions. I look forward to working with you and the reviewers to move this manuscript closer to publication in *PLOS ONE*.

The manuscript has been rechecked and the necessary changes have been made in accordance with the reviewers’ suggestions. The responses to all comments have been prepared given below.

Thank you for your consideration. I look forward to hearing from you.

Sincerely,

Xinli GAN

Tel: +86-18275239823

Fax:+86-0851-88195014

ganxinli2007@126.com

**Additive list**

**To reviewer #1:**

**Introduction :**

**Comment 1:** First paragraph, 3rd line from bottom – I do not think “rational” is the word you want there. Suggest replacing with “sustainable.”

**Answer:** The manuscript has been thoroughly checked, and the mistakes have been fixed with the help of a native English speaker. We have revised this sentence as follows: “Therefore, the development of an effective and sustainable way of using crop straw has become an important research and real-world application issue.”(Line 32)

**Comment 2:** First paragraph, sentence starting with “One possible method….” This is a sentence fragment/improper tenses/punctuation. It needs to be rewritten.

**Answer:** We have revised this sentence as follows: “Therefore, the development of an effective and sustainable way of using crop straw has become an important research and real-world application issue.”(Line 32)
**Comment 3:** Second paragraph, first sentence – remove the words “owing to repeated usage.” They are unnecessary, and the sentence should just start with “The performance of asphalt pavements….”

**Answer:** We have revised this sentence as follows: “The performance of asphalt pavements inevitably degrades under the combined action of loads and the natural environment.”(Line 34)

**Comment 4:** Second paragraph, sentence that starts with “Under” – the word “owing” is used incorrectly. You mean “because of”

**Answer:** We have revised this sentence as follows: “The performance of asphalt pavements inevitably degrades under the combined action of loads and the natural environment..”(Line 34)

**Comment 5:** Next sentence, remove “Being a cementitious material”

**Answer:** We have revised this sentence as follows: “Asphalt ages under the influence of light and temperature; this reduces its cementation ability and negatively affects the durability of asphalt pavements [8].” (Line 38)

**Comment 6:** Sentence after that, remove the words “Therefore, owing” and replace with “Because of”

**Answer:** We have revised this sentence as follows: “Owing to the high cost and environmental sensitivity of asphalt pavements, researchers are focusing on improving their durability.”(Line 40)

**Comment 7:** Third paragraph – extra “the” before adding.

**Answer:** We have changed "adding" to "the addition of"(Line 42)

**Comment 8:** Third paragraph, third line – remove the comma after mixtures.

**Answer:** We have removed the comma after “mixtures” and revised this sentence as follows: “and aging resistance of asphalt and asphalt mixtures and does not adversely affect other asphalt pavement performance indicators [9, 10].”(Line 44)

**Lit Review :**

**Comment 1:** 4th line – remove the word “purposes” after “energy generation;” remove the dashes around the “such as” clause and replace with commas.

**Answer:** We have revised this sentence as follows: “Doyle et al. [12] studied the economic benefit of the use of straw as a fuel, as raw material for papermaking, and as animal feed, compared to burning the straw in a field.”(Line 56)

**Comment 2:** Sentence that starts with S.M. Shafie – remove the comma after “logistical costs.”

**Answer:** We have changed this sentence to “[Shafie](https://www.sciencedirect.com/science/article/pii/S0360544213004969#!) et al. [14] investigated the economic feasibility (i.e., operating, capital, and logistic costs) of rice straw co-firing at coal power plants in Malaysia and determined that co-firing rice straw in an existing coal power plant could reduce CO2 emissions.” (Line 59)

**Comment 3:** Same sentence and next sentence – inconsistent formatting with your references. Above you were referring to your references by last name only. Then you did initials and last name. And here it is full first name and last name (Mingxing Sun). And then in the next sentence you are back to last name only (Nair). Which is correct? Be consistent and stick to it!

**Answer:** We have revised the format of the references.(Line 62)

**Comment 4:** Next paragraph – same inconsistencies noted above (Zhao Sheng or just “Sheng” would probably be more correct); Renaldo Walters vs.”Walters.” Etc.

**Answer:** We have revised the format of the references.(Line 76)

**Comment 5:** Sentence that starts with “Renaldo Walters” – you wrote 3 and 6%. The correct punctuation is “3% and 6%.”

**Answer:** We have added "%" after "3"(Line 82)

**Comment 6:** Sentence starting with “FU Zhen” – this sentence does not make any sense unless you add the word “at” before the word “were.” (i.e. …asphalt that were conducted at….”

**Answer:** We have revised this sentence as follows: “Zhen et al. [25] prepared biochar-modified asphalt via the high-speed shear method and conducted laboratory rolling thin film oven tests and 60 °C dynamic viscosity tests of the biochar-modified asphalt; they found that biochar could increase the bond capability and resistance to flow deformation of the modified asphalt.”(Line 84)

**Materials and Methods :**

**Comment 1:** Sentence right after Fig. 2 – this should be in the past tense. I.e., the preparation process was as follows.

**Answer:** We have revised this sentence as follows: ”The preparation process was as follows”(Line 107)

**Comment 2:** Step 4 – the subject of this sentence is “the straws.” Therefore, the correct pronoun to refer to this subject is “they” and not “it.”

**Answer:** We have revised this sentence as follows: ” After cooling, the bio-char was ground to a powder using a grinder.”(Line 114)

**Comment 3:** Section 4.3 – What are the details associated with your physical property testing? Were there repetitions? If so, how many? Was a standard followed? If so, it must be referenced.

**Answer:** These tests followed the Standard Test Methods of Bitumen and Bituminous Mixtures for Highway Engineering (JTG E20-2011) of China. This has been specified in the manuscript. Following these standards, the ductility, softening point, and penetration tests were repeated three, two, and three times in each group, respectively. (Line 149)

**Results and Discussion :**

**Comment 1:** Figs. 3 & 4 – I can see from the figure that the authors did not bother to make a “clean” document for submission and instead left the track changes on in MS Word. This is sloppy/one should expect better than this if this paper is truly ready for publication.

**Answer:** We are sorry for these problems. We have carefully proofread the manuscript and fixed all formatting issues.(Line 162)

**Comment 2:** Section 5.2 – third paragraph through the end of this section – I can clearly see the point the authors are making – bio-char has much smaller particles statistically than commercial-grade charcoal. However, I find myself wondering if this is the best way to present these data. Would it not be more efficient/concise to express Table 4 in graphical form similar to an aggregate particle distribution chart or a geotechnical soil particle chart? I would think this would make the data a little bit easier to read/interpret. In either case, remove the word “owing” in the 3^rd^ to last line from the bottom of this section. Almost always, you can strike “owing” from your writing and either remove it entirely or replace it with the word “because.”

**Answer:** Given the small size of the carbon powder particles (micron size), it is difficult to characterize them by the grading distribution diagram. Instead, we performed a laser particle size analysis test of the crop straw bio-char and commercial charcoal to evaluate the particle size characteristics of the carbon powder. As suggested, we replaced the word “owing” with the word “because.” (Line 187)

**Comment 3:** Section 5.3 – remove the word “moreover” from the sentence that starts with “As the biochar content increased…” Also add semicolon before “and the ductility”

**Answer:** We have revised this sentence as follows: “It was found that, with the increase in bio-char content, the asphalt penetration decreased, the softening point increased, and the ductility decreased.”(Line 228)

**Comment 4:** Same sentence in Section 5.3 – you said that bio-char “significantly increased high-temperature performance.” Be careful with the word significantly unless you really mean it. It does not appear that there was any testing repetition, although based upon Section 4.3, it is very difficult to tell exactly what the authors did. If there were no repetitions, it would be very difficult to prove statistical significance. If the authors would be willing to couch their argument to something less definitive (i.e., remove the word “significant”) this would be fine as well. In either case, I would think a graphical presentation might be a better way to present these data as opposed to putting them in tabular form. Doing so would allow you to do much more interesting analysis like (for example) plotting a regression line through the data points. This does not prove statistical significance, but it does show a relationship between % biochar and physical properties and would be much more sophisticated than what is presented here. I just plotted the data myself and noted that there appears to be a linear relationship between softening and % bio-char; and apparent exponential relationships between penetration & density and % bio-char. Each of these relationships had relatively high R-squared values.

**Answer:** As shown in many previous studies, the softening point, penetration, and ductility of asphalt are significantly related to the high temperature performance of asphalt. Higher softening point and lower penetration and ductility would lead to a better high temperature performance of the asphalt. For these tests, we followed the Standard Test Methods of Bitumen and Bituminous Mixtures for Highway Engineering (JTG E20-2011) of China. Each test was repeated in accordance with the requirements of the standard.(Line 100, 149, 155)

**Conclusions :**

**Comment 1:** The authors have dutifully summarized there work here. However, they have done almost nothing to connect the dots for the reader. In other words, what do these results imply for future asphalt mixtures? Might they be applicable to other types of asphalt with different aggregate?

**Answer:** We rewrote the conclusions as follows: “We confirmed that biochar can be prepared from crop straw and can be added into asphalt and found that the addition of crop straw biochar significantly improved the high-temperature performance of asphalt. Although the addition of crop straw reduces the low-temperature performance of asphalt, there is hardly any low-temperature road failure in high-temperature regions; thus, the low-temperature performance requirement of asphalt is very low. The addition of crop straw biochar was shown to increase the high-temperature performance of asphalt, which is beneficial to asphalt pavement in high-temperature regions. The crop straw biochar and commercial coal mainly consist of C, O, Si, and K, indicating that the biochar produced from crop straw has properties similar to commercial coal. Based on experimental results, we determined the appropriate amount of straw biochar powder to be used in asphalt to be 6%.”(Line 263)

**To reviewer #2:**

**Comment 1:** The abstract states that “viscosity of the asphalt binder modified using different dosages of biochar powder were measured”. However, the viscosity results are presented for the binder with 6% biochar only. Please make suitable corrections to the abstract.

**Answer:** We have revised this sentence in the abstract as follows: ”and apparent viscosity of the asphalt binder with 6% bio-char were measured at 120, 135, 150, 160, and 175 °C.”(Line 15)

**Comment 2:** No results of technical performance (penetration, softening point, ductility, viscosity) are presented for commercial charcoal modified asphalt binders. Please provide an objective statement, preferably in Section 1 of the manuscript.

**Answer:** Previous studies on charcoal-modified asphalt used charcoal prepared from wood in the laboratory or commercial charcoal. We have specified this in Section 1.(Line 46)

**Comment 3:** The authors have reviewed literature on the use of biochar for asphalt binder modification. I suggest the authors to also include the following relevant articles for an updated state-of-the-art on the subject:
(1) Wu, Y., Cao, P., Shi, F., Liu, K., Wang, X., Leng, Z., ... & Zhou, C. (2020). Modeling of the Complex Modulus of Asphalt Mastic with Biochar Filler Based on the Homogenization and Random Aggregate Distribution Methods. Advances in Materials Science and Engineering, 2020.
(2) Kumar, A., Choudhary, R., Narzari, R., Kataki, R., & Shukla, S. K. (2018). Evaluation of bio-asphalt binders modified with biochar: a pyrolysis by-product of Mesua ferrea seed cover waste. Cogent Engineering, 5(1), 1548534.

**Answer:** We have carefully read these two articles and cited them in our paper.(Line 354–358)

**Comment 4:** Was an oxygen-deficit environment upheld during the generation of biochar? If yes, then it should be reported that the biochar was prepared under pyrolytic conditions.

**Answer:** Indeed, the biochar was prepared under pyrolytic conditions. The muffle furnaces were sealed to maintain an oxygen-deficit environment. We have specified this in the section 3 ("Materials and Methods”). (Line 113)

**Comment 5:** Did the authors observe any foaming of the asphalt binder on addition of biochar at the mixing temperatures?

**Answer:** Indeed, foaming of the binder was observed. Once the carbon powder is mixed into the asphalt, the air present in the pores of the biochar powder enter the asphalt, causing the asphalt to produce some foam.

**Comment 6:** Placement under 120 °C for 6 hours will likely lead to an accelerated aging of the modified blend. Can authors justify this step? Were any arrangements made to ensure that the control binder also underwent a similar degree of aging before being subjected to testing?

**Answer:** At high temperature, the aging of asphalt is inevitable. In fact, any modification of the asphalt will inevitably lead to asphalt aging to a certain extent. Even the asphalt stored in asphalt tanks above 120 ℃ (which is common before construction) ages. At present, there is no better way to prevent asphalt from aging.

**Comment 7:** Authors are encouraged to discuss the implications of SEM and EDS results. How can the morphological features of biochar affect the asphalt-biochar interaction?

**Answer:** We have discussed some analysis results in the manuscript: When the powder is added into the asphalt binder, the asphalt gets wrapped on the surface of the powder particles, and interfacial tension is induced owing to the combined action of intermolecular forces between the two phases, chemical bonds, and mechanical locking forces. Under the action of the interfacial tension, the asphalt will bond, thus reducing the plasticity of the asphalt. Finer carbon particles lead to larger surface area and stronger adsorption of asphalt.(Line 220)

**Comment 8:** Section 5.2: It is reported that “…biochar powder is more likely to agglomerate because of interfacial tension”. Since the interfacial tension was not measured in the study, please support this statement with appropriate reference(s).

**Answer:** The expression of this sentence was indeed inaccurate and has been revised as follows: “With equal quality, the crop straw biochar powder has a larger contact area with asphalt, so it mixes less than the commercial charcoal. Some studies have shown that smaller-sized particles (especially those smaller than 150 µm) form stronger agglomerates [30]. This is because the van der Waals force between the particles is strong when the particles are small, which induces the particles to agglomerate [31]. Owing to its smaller particle size, the crop straw biochar powder agglomerates more easily; hence, the crop straw biochar-modified asphalt requires a longer shearing time during preparation. When the powder is added into the asphalt binder, the asphalt gets wrapped on the surface of the powder particles, and interfacial tension will be induced under the combined action of the intermolecular force between the two-phase, chemical bond, and mechanical locking force. Under these effects, the plasticity of asphalt will be reduced, and deformation does not occur easily under the action of the force [32].”(Line 215)

**Comment 9:** It is reported that biochar particles passing the 75-micron sieve were used in the study. However, Figure 5 shows some amount of biochar particles near the 1000-micron size. An explanation is needed for this observation. Could it be due to possible contamination by larger particles during the analysis?

**Answer:** We used biochar particles that passed a 75-micron sieve. However, owing to the large number of carbon powder particles, it is inevitable that some particles greater than 75 microns will be mixed into the asphalt.(Line 198)

**Comment 10:** At several instances, the authors have emphasized that C content in biochar was found higher than charcoal. Please indicate what does it mean in physical terms: does it have a bearing on the asphalt-biochar blend properties?

**Answer:** Carbon is stable in asphalt. The results show that the carbon content of biochar is higher than that of charcoal, which indicates that the application of biochar in asphalt modification is feasible. A higher carbon content will not adversely affect the performance of asphalt.

**Comment 11:** Some minor comments are:
o Replace ‘density’ with ‘ductility’ in Table 5 and also correct the units.
o Replace ‘brockfield’ with ‘Brookfield’ in Section 4.3.
o Replace ‘aggregation’ with ‘aggregates’ in Section 5.3.

**Answer:** We have corrected these errors in the revised manuscript.(Line 155,228,245)

**To reviewer #3:**

**Comment 1:** Is not there any anther usage from strew waste materials? Recycling or in animal food science? It seems that the authors claimed that the only option, except land-filling, is to use it as asphalt material reinforcement agent! Please double check and verify your claim.

**Answer:** There are many ways to use crop straw apart from asphalt modification. We mentioned some methods in the introduction.(Line 56)

**Comment 2:** In figure 1, as far as I know, such straw is good for sheep grazing.

**Answer:** China is a large agricultural country, so large quantities of straw are produced in the cultivation of crops. If sheep grazing is adopted, the treatment of these straws will be very slow. Moreover, many types of straws, such as corn straw, cannot be consumed by sheep. In addition, there are not enough sheep to consume all straw produced in crops. A statistical analysis showed that in Chinese mainland only, the amount of straw burned every year is as high as 1.4 billion tons.(Line 29)

**Comment 3:** In table 1, specify the viscosity measurement temperature.

**Answer:** The viscosity was measured at 135 ℃; this has been specified in Table 1.(Line 104)

**Comment 4:** What is the advantage of providing elements of biochar straw? Once the authors have taken molecular dynamic simulation or investigate the physiochemical interaction between asphalt binders and modifier, providing such information would be beneficial.

**Answer:** The elements in the crop straw bio-char are mainly C, O, Si, and K, which is consistent with the chemical composition of commercial charcoal. This indicates that the bio-char produced from crop straw has a similar properties to commercial charcoal. (Line 185)

**Comment 5:** The dosage shown in Table 5, is based on the total weight of binder? If so, please alluded it.

**Answer:** We have added a table, so the original table 5 is now table 6. The dosage shown in Table 6 refers to the percentage by mass of asphalt binder. This has been specified in the manuscript.(Line 228)

**Comment 6:** Based on old fashion test result, it is hard to believe that such agent would be beneficial for asphalt industry. To understand better, if you add any solid garbage in the asphalt binder, the same trend would be perceived! The advanced rheological test result would help to distinguish the different and make it advantageous be cleared.

**Answer:** This article aims to provide an application method for crop straw. Given the large amount of asphalt used for pavement paving, adding crop straws in the form of biochar into asphalt can consume a large amount of straw. Although the biological addition of crop straw reduces the low-temperature performance of asphalt, there is hardly any low-temperature road failure in high-temperature regions; thus, the low-temperature performance requirement of asphalt is very low. The addition of crop straw bio-char increases the high-temperature performance of asphalt, which is beneficial to asphalt pavement in high-temperature regions. (Line 264)

**To Additional Editor：**

-For this and ALL future submissions, line numbers need to be added to the manuscript and changes made need to be specific by line number
**Answer:** We've added line numbers to the manuscript.

-Abstract needs to do a better job reporting novel results: specifically, HOW did addition of crop straw bio-char significantly improve the high-temperature performance? How did performance compare with commercial charcoal?

**Answer:** We discussed the results of the penetration, softening point, and ductility tests of commercial charcoal-modified asphalt with different carbon powder contents. It was found that the influence of biochar and commercial charcoal on the high temperature performance of asphalt is similar. (Line 228)

- Change all ‘bio-char’ to ‘biochar’

**Answer:** We have changed the term ‘bio-char’ to ‘biochar’ throughout the manuscript.

-Could use more editing from native speaker. It is hard to believe that this was done given the awkwardness of language throughout the text. For example, tense use must made be consistent throughout manuscript
**Answer:** We have re-edited the manuscript to improve the language.

-For goodness sake, please stop answering every reviewer comment with: “ Thanks for your very thoughtful suggestion”
**Answer:** We have deleted these expressions.

-‘ et al.’ cannot be used in the author lists in the bibliography
**Answer:** We have re-edited the format of the references.

-Comments made by reviewers, should not only be responded to in the text (not just in comments back to reviewers). For example, when a review asked “Figure 5 shows some amount of biochar particles near the 1000-micron size.”, response/explanation should be made in the manuscript

**Answer:** We have added the following description in the manuscript: From Fig 3, it can be seen that some biochar particles have a size in the millimeter range; this is inevitable given the large number of carbon particles. Nevertheless, the amount of such large particles is very small and can be neglected.(Line 198)

-statistical significance needs to be considered in the text. For example, it the C content of the biochar significantly different from that of charcoal? Are you presenting averages? Need std. dev. This is true of ALL your parameters examined. Also, correct significant figures need to be correct and consistent

**Answer:** We have used the method of Single Factor ANOVA in Microsoft Excel software to analyze the difference in the element content of the two kinds of charcoal. We have mentioned this in the revised manuscript. The results of this analysis showed that there was no significant difference in the element content between the two kinds of charcoal. (Line 228)

In Table 1, the number decimal places in the values of each parameter is chosen based on the requirements of Standard test methods of bitumen and bituminous mixtures for highway engineering (JTJ E20-2011). In other tables, the same decimal places are reserved for the data in the same table.

-Your first paragraph needs improvement. Incineration is NOT a straw production method. Also, incineration is certainly not the most common use of straw. How much straw is actually incinerated?
Related to this, I and reviewer 3 think you are mistakenly representing straw as something that needs to be gotten rid of. Is this the case? We doubt it. Please address in the conclusion, whether, given the minor benefits, this is really the best use of straw. Perhaps other asphalt additives that are truly waste would be better put to use here.

**Answer:** A statistical analysis showed that in Chinese mainland only, the amount of straw burned every year is as high as 1.4 billion tons.(Line 29)

Straw can have many applications, depending on the type. Most of the existing utilization methods have specific requirements for straw. For example, the palatability of straw is important for preparing straw into feed. Not all types of straw are eaten by animals, and the use of straw to prepare chemical products has high cost. In this study, we produced biochar powder from straw and used it to prepare asphalt pavement. The advantage of this method is that it is simple and applicable to all straw.

-Delete figure 1 and 2. Everyone knows what straw and charcoal looks like.
**Answer:** We have deletes figures 1 and 2.

- Figures need to be numbered and ordered consecutively
**Answer:** We have changed the Figure numbers.

-Fig. 6 has only two data points. This cannot make a correlation. It should be deleted along with all mention of a linear relationship

**Answer:** We discussed the viscosity experiment results of asphalt at 120, 150, and 160 ℃, and established the relationship between viscosity and temperature by using the experimental data obtained at five temperatures. These contents have been added in the manuscript.(Line 256)

-Caption on figure 4 insufficiently describes the image
**Answer:** Following your suggestion, we have deleted Figures 1 and 2 in the manuscript and changed the original figure 4 number to figure 2. We added the following description of Figure 2 to the manuscript: “In Fig 2, carbon, silicon, oxygen, and potassium are marked in red, blue, green, and pink, respectively. It can be seen that both types of carbon powder mainly consist of carbon and contain some silicon, oxygen, and potassium. The element distribution of biochar is more uniform than that of commercial charcoal” (Line 170)

-ALL your asphalt data tables (tabs. 1 and 5), need to compare need to compare the properties of asphalt, with that of asphalt with biochar and asphalt with commercial charcoal. Otherwise, there is little value in this research and I will have to reject the manuscript.
**Answer:** We reported the results of the penetration, softening point, and ductility tests of commercial charcoal-modified asphalt with different carbon powder contents in Table 6 (Line 228). Table 1 shows the technical indexes of matrix asphalt, that is, the technical indexes of the raw materials used to prepare the modified asphalt.

Reviewer #3 Comment 4: What is the advantage of providing elements of biochar straw? Once the authors have taken molecular dynamic simulation or investigate the physiochemical interaction between asphalt binders and modifier, providing such information would be beneficial.
Answer: Thanks for your very thoughtful suggestion. The elements in the crop straw bio-char are mainly C, O, Si, and K, which is consistent with the chemical composition of commercial charcoal. This indicates that the bio-char from crop straw has a similar effect to commercial charcoal.
Editor: I don’t find this answer sufficient. Elemental composition is not an advantage. The abstract states: “bio-char significantly improved the high-temperature PERFORMANCE of asphalt”. This need to be more clear in abstract and conclusion.
**Answer:** We have modified the summary and conclusion sections to make it clear that the addition of biochar to asphalt can improve the high temperature performance of asphalt.(Line 264)

- The dosage shown in Table 5 refers to the percentage by mass of asphalt binder. – should be added to the Table
**Answer:** We have replace "dosage" with "mass percentage of carbon powder content in asphalt binder"(Line 228)

-The ‘binder’ needs to be specified in the manuscript.

**Answer:** We have specified the preparation location and technical performance of the asphalt binder used in this study.(Line 100)
